# Reliability of Fronto–Amygdala Coupling during Emotional Face Processing

**DOI:** 10.3390/brainsci9040089

**Published:** 2019-04-19

**Authors:** Camilla L Nord, Alan Gray, Oliver J Robinson, Jonathan P Roiser

**Affiliations:** 1 MRC Cognition and Brain Sciences Unit, University of Cambridge, Cambridge CB2 7EF, UK; 2 Institute of Cognitive Neuroscience, University College London, London WC1N 3AZ, UK

**Keywords:** reliability, functional magnetic resonance imaging (fMRI), connectivity, emotion processing, amygdala, prefrontal cortex

## Abstract

One of the most exciting translational prospects for brain imaging research is the potential use of functional magnetic resonance imaging (fMRI) ‘biomarkers’ to predict an individual’s risk of developing a neuropsychiatric disorder or the likelihood of responding to a particular intervention. This proposal depends critically on reliable measurements at the level of the individual. Several previous studies have reported relatively poor reliability of amygdala activation during emotional face processing, a key putative fMRI ‘biomarker’. However, the reliability of amygdala connectivity measures is much less well understood. Here, we assessed the reliability of task-modulated coupling between three seed regions (left and right amygdala and the subgenual anterior cingulate cortex) and the dorsomedial frontal/cingulate cortex (DMFC), measured using a psychophysiological interaction analysis in 29 healthy individuals scanned approximately two weeks apart. We performed two runs on each day of three different emotional face-processing tasks: emotion identification, emotion matching, and gender classification. We tested both between-day reliability and within-day (between-run) reliability. We found good-to-excellent within-subject reliability of amygdala–DMFC coupling, both between days (in two tasks), and within day (in one task). This suggests that disorder-relevant regional coupling may be sufficiently reliable to be used as a predictor of treatment response or clinical risk in future clinical studies.

## 1. Introduction

Measurement reliability is essential when translating research findings to clinical practice. Any study that describes brain function associated with risk or resilience to developing a neuropsychiatric disorder or with good or poor treatment outcome following treatment, rests on the assumption that the measurement has adequate reliability. For example, if a study finds that depressed patients who respond to fluoxetine have higher subgenual anterior cingulate cortex (sgACC) activation than non-responders, assessed with functional magnetic resonance imaging (fMRI), this could only be of clinical use if the measurement of each patient’s sgACC activation were reliable, such that the clinical prognosis derived from the scan of an individual patient was consistent over time. Only then could brain activation (in the sgACC in this example) be tested as a putative fMRI ‘biomarker’ to predict clinical response. 

Recently, we conducted a test–retest reliability analysis of activation for three commonly proposed biomarkers for depression, the left and right amygdala and the sgACC, measuring fMRI activation during three different emotional face paradigms [1]. Discouragingly, we found consistently poor reliability across all three tasks, both across days (two weeks apart) and within the scan session (two runs 15 minutes apart). This replicated some previous poor reliability findings in the amygdala [2], although others have produced more optimistic estimates [3,4]. 

Several recent studies have suggested various measures of functional connectivity—i.e., the covariation of fMRI signal between two or more brain regions over time—as putative ‘biomarkers’. For example, studies have reported a relationship between functional connectivity and ketamine response in depression [5], antipsychotic response in schizophrenia [6], response to brain stimulation (repetitive transcranial magnetic stimulation, rTMS) in depression [7], and cognitive behavioural therapy (CBT) response in anxiety [8]. However, the reliability of these connectivity measures has not been comprehensively tested. In this paper, we therefore explore the reliability of a specific connectivity measure in the same dataset we analysed previously.

The example we focus on in this manuscript is a cortical–subcortical circuit that may play an important role in threat processing [9], a behaviour with transdiagnostic relevance across affective and anxiety disorders [10]. This circuit involves a region that encompasses areas of the dorsomedial frontal/cingulate (DMFC) cortex, which shows increased connectivity with the amygdala during both induced and pathological anxiety [9,11]. A large body of evidence in humans and rodents implicates the extended amygdala in aversive processing [12,13,14,15], a key cognitive mechanism in anxiety disorders [16]. However, the amygdala functions in concert with more frontal (‘higher-order’) neural regions. There is substantial evidence suggesting that anxiety-related processes, in fact, reflect a bidirectional modulation between amygdala and medial prefrontal circuitry, in particular the DMFC [16,17], which itself has been shown to be overactive in pathological anxiety [18]. Nonhuman primate research has found that amygdala–DMFC coupling is involved in fear learning and other anxiety-related behaviours [16,19,20]. Moreover, this coupling can be down-regulated by attentional instruction, suggesting that psychological therapy might function via similar neural mechanisms [21]. Therefore, DMFC–amygdala coupling is a crucial potential biomarker of anxiety and, potentially, also of therapeutic response [16,22].

We built on our previous work by examining the test–retest reliability of task-modulated covariation of fMRI signal between our initial ‘biomarker’ regions (the left and right amygdala and the sgACC) and the DMFC target region derived from a circuit implicated in anxiety [9]. Specifically, we tested whether the psychophysiological interaction (PPI, i.e., the modulation of coupling by a psychological factor, in this case, processing emotional faces) between the amygdalae and sgACC seed regions and the DMFC target was reliable within an individual. We examined reliability across scan days two weeks apart, as well as within the same scanning session across two runs of the task. 

## 2. Materials and Methods 

This re-analysis was performed on a previously acquired dataset; the initial fMRI analysis was described in more detail in our earlier publication [1]. In summary, we extended the initial task-related *activation* analysis to encompass *connectivity*. The key details are re-stated below for completeness.

### 2.1. Participants

We recruited healthy controls (*N* = 29, age range 18–40, mean age 26 (*SD* = 6.24), 10 male) through the UCL Institute of Cognitive Neuroscience and Department of Psychology subject databases. We included only right-handed fluent English speakers (meeting standard MRI safety criteria) without a recent (six-week) history of illegal substance use or any history of neurological or psychiatric disorders (the latter was screened by using the Mini International Neuropsychiatric Interview, version 5.0.0 [23]). We initially recruited 35 participants: 4 of them did not attend their follow-up fMRI scan, and data loss occurred for 2 of them.

The study was approved by the UCL Departmental Research Ethics Committee (ID: fMRI/2013/005). The participants provided informed consent in accordance with the Helsinki Declaration and were compensated £30 for taking part. 

### 2.2. Protocol

Our experimental protocol involved three separate testing days. The participants first attended a screening session at the UCL Institute of Cognitive Neuroscience to determine eligibility and complete practice versions of the fMRI tasks. The second and third sessions took place at the Birkbeck-UCL Centre for Neuroimaging 9–21 days later (mean 14.33, *SD* = 2.10). On each of these scan sessions, the participants performed three tasks twice in the scanner (order counterbalanced between participants, with order kept constant within each participant), using an MR-compatible button box. 

The tasks have been described in detail previously [1] (see Table 1 for summary). Briefly, the emotional identification (EI) task involved explicitly judging the emotion (happy, fearful, or neutral) of sixty face stimuli (task adapted from a separate study [11]). Two participants were excluded from this task analysis because of a high proportion of non-responses (>20 trials). The face matching (FM) task [24] consisted of 30 trials (split into five blocks) of either shape matching (18 trials, 3 blocks) or face matching (12 trials, 2 blocks). In both cases, the participants were instructed to match a centrally presented emotional face or centrally presented shape (both displayed for 5 s) with one of two alternatives displayed at the bottom (two test faces or two test shapes). No participants were excluded because of poor task performance. In the gender classification (GC) task [25], the participants were instructed to classify the gender of emotional faces (displayed for 2 seconds, happy, fearful, or neutral faces, separated into blocks; equal proportion of male and female faces, displayed in a random order). Each emotion block occurred 4 times per run (12 blocks total, 8 stimuli per block). One participant (with performance worse than chance) was excluded from this analysis. 

### 2.3. Image Acquisition and Analysis

fMRI image acquisition has been described in detail previously [1]. Briefly, gradient-echo T2*-weighted images were acquired using a Siemens Avanto 1.5 Tesla MRI scanner employing a 32-channel head coil (this head coil improves signal-to-noise ratio up to 3.5 times compared to standard 8- or 12-channel coils [26]). We collected 36 slices per volume, slice gap 1 mm (2 mm slices; 50% distance factor) (see Table 1). The echo planar imaging (EPI) sequence was highly optimised to minimise ventral prefrontal cortex dropout (for extensive sequence details, see previous work [1,25,27]): echo time = 50 ms, repetition time/slice = 87 ms, slice thickness = 2 mm, in-plane resolution = 2 × 2 mm. One fieldmap per participant per day was acquired using identical parameters to the EPI scans; for each participant, we also acquired one magnetization-prepared rapid gradient-echo T1-weighted 1 mm isotropic anatomical scan.

We used Statistical Parametric Mapping (SPM12; Wellcome Trust Centre for Neuroimaging, London, UK, www.fil.ion.uck.ac.uk/spm) in Matlab R2015a (for the initial region-of-interest (ROI)-based analyses) and Matlab R2018a (for the PPI analyses). All data were slice-time corrected to account for the long repetition time (TR) (3.132 seconds). Preprocessing was identical to that described previously [1]. 

### 2.4. Psychophysiological Interaction Analysis

We used these data to conduct a PPI analysis, a functional connectivity method commonly applied to task-based fMRI data. This analysis reveals how experimental task conditions modulate the covariation in signal between a ‘seed’ region and the rest of the brain [28]. This is in contrast to the so-called “resting-state” functional connectivity, which examines such covariation independent of any specific cognitive demands. Therefore, PPI analysis can reveal which voxels in the brain exhibit different covariation with the seed region as a function of task condition [28] (in our study: for GC and EI, faces > fixation cross; for FM, faces > shapes). Note that PPI analyses do not provide any inferences about the directionality of this relationship or whether any connections are direct or indirect.

We followed the steps of a PPI analysis outlined by O’Reilly and colleagues [28]. We first defined anatomical masks for our three seed regions (PickAtlas left and right amygdala masks and a custom-made sgACC mask described previously [1]; note the sgACC mask was bilateral, to adhere with typical practice in the field and maintain comparability between these and our previous results [1]) and extracted representative time courses for each seed region. One subject did not show any suprathreshold activation in the left amygdala seed on any of the tasks; for this subject, we analysed only the right amygdala and the sgACC. Next, we computed the PPI time course (a single regressor describing the interaction between the time course of each region and the time course of each task). Finally, we entered this interaction regressor into a general linear model which included the physiological and psychological time courses as covariates. All models also included six movement regressors of no interest, and for the gender classification task, an error regressor only in those participants who made errors. For each task, we constructed parameter estimate images for the primary contrast of interest (for gender classification and emotion identification, faces > fixation cross, the implicit baseline; for emotion matching, faces > shapes). The results (voxels where activation was significantly explained by the interaction between seed and task time course) therefore describe only those voxels where the variance explained was over and above (1) the physiological covariation between each voxel and the seed region, and (2) the haemodynamic response function (HRF)-convolved time course of the task [28]. 

**Figure 1:**
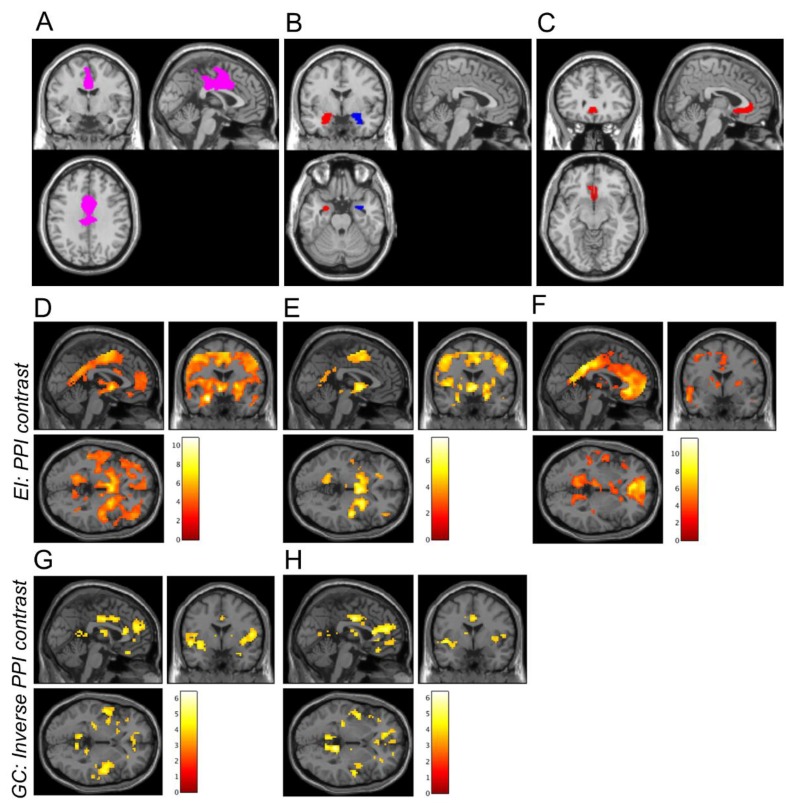
A priori ROIs and whole-brain PPI connectivity averaged across all runs and scan days. Figure A depicts the DMFC ROI used as a target region in the PPI analysis (**A**). Figure B,C depict the three seed regions: the left and right amygdala (**B**) and the subgenual anterior cingulate cortex (**C**). D,H depict results from seed regions showing significant PPI effects in the DMFC cortex ROI. Increases in whole-brain connectivity (positive PPI contrast) were observed during the EI task for the left amygdala (**D**), right amygdala (**E**), and subgenual anterior cingulate cortex (**F**) seeds. Decreases in whole-brain PPI connectivity (negative PPI contrast) were observed during the GC task for the left amygdala (**G**) and right amygdala (**H**) seeds. For illustrative purposes only, images were thresholded at *p* < 0.001 (uncorrected), and the colour bars indicate t-values. The inverse PPI contrast is displayed for the GC task (G,H) because mean PPI connectivity with the DMFC ROI was significantly negative (see Figure 2).

We performed a PPI analysis for each region (*N* = 3) and each task (*N* = 3). We took an a priori ROI approach to the analysis. Our a priori ROI was defined as the more rostral of two functionally defined ROIs created from clusters emerging from a whole-brain threat-by-valence interaction during a threat of shock task [11] and included, primarily, areas of DMFC (see Figure 1A; see Figure 1B,C for amygdala and sgACC masks, respectively). Our ROI analysis approach proceeded in much the same way as the ROI analyses in our previous paper, but now the extracted betas represented the modulation of covariance between the ROI and the seed by the task, rather than activation within the ROI.

Across an average of all four runs of each task (two on each day), we conducted one-way t-tests to verify there was a significant PPI effect between these regions. We then selected the regions showing a significant effect to perform reliability testing. We also tested for effects of day, run, and the interaction between day and run, using an analysis of variance on each ROI for each task. 

### 2.5. Reliability Testing

To quantify within-subject reliability, we performed intraclass correlation coefficient (ICC) analyses on the PPI results, using the Statistical Package for the Social Sciences 22 (http://www.ibm.com/analytics/us/en/technology/spss/). The ICC enabled us to quantify the stability of measurement across each fMRI session [29] and is usually interpreted as a ratio of between-subjects to within-subjects variance [30] (an ICC = 1.0 would indicate perfect agreement between test and retest fMRI sessions). For each seed region (left and right amygdala; sgACC), we calculated five ICCs: three between-day ICCs (averaged across both runs, as well as separately for the first and second runs) and two within-day ICCs (between the two runs, separately for each day). 

We employed a two-way mixed effects ICC [31], defined as:

ICC(3,1) = BMS - EMS/BMS + (k - 1)*EMS

This form of the ICC has three important characteristics: it assumes the effect of measure (scanner) to be fixed, the effect of participant to be random, and tests for “consistency” (rather than “absolute” agreement) between sessions. We report average measures (rather than single measures) ICCs. 

We adhered to a conventional interpretation of ICCs: ICC < 0.4 was interpreted as poor reliability; ICC = 0.4–0.75 as moderate-to-good reliability; ICC > 0.75 as excellent reliability [2,32]. We also report p-values and 95% confidence intervals obtained from an F-test against the null hypothesis. 

### 2.6. Power Analysis

Our power analysis was computed for the original reliability dataset. To achieve 80% power to detect a moderate-to-large effect size (correlation r = 0.5, which we chose as a clinically meaningful degree of reliability) at our specified alpha (0.05, two-tailed), we required 26 participants [1]. 

## 3. Results

### 3.1. PPI Analysis

**Figure 2:**
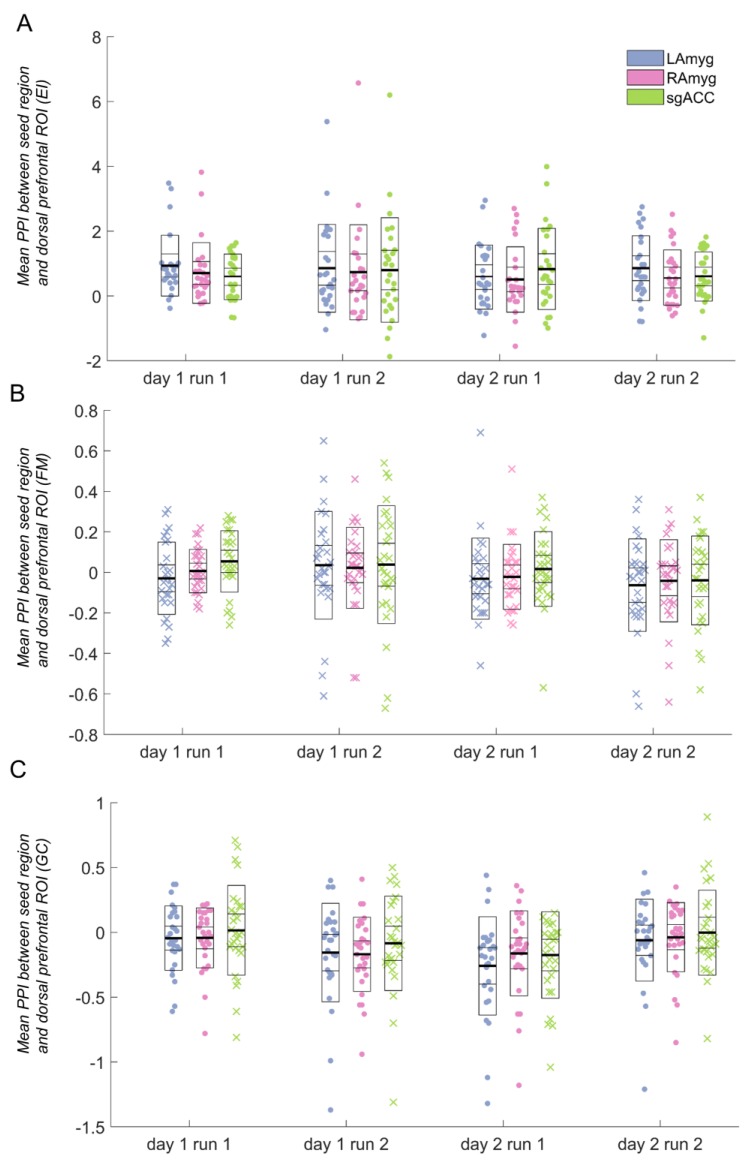
Psychophysiological interaction (PPI) parameter estimates for each task between the seed regions and the dorsomedial frontal/cingulate (DMFC) cortex region of interest. Data marked with “x” (as opposed to those marked with a filled circle) indicate runs that were not significantly different from zero (and therefore were not analysed further). Horizontal lines in the box plots indicate the mean (darkest line), standard deviation, and 95% confidence interval. A: Emotion identification (EI) task; B: Face matching (FM) task (not analysed further, as the average PPI parameter estimates were not significantly different from zero); C: Gender classification (GC) task (the subgenual anterior cingulate cortex (sgACC) seed was not analysed further, as the average PPI parameter estimate was not significantly different from zero). ROI: region of interest; LAmyg: left amygdala; RAmyg: right amygdala.

We first analysed the overall PPI effects (collapsing across day and run); we report average PPI effect size (Cohen’s d, or standardised mean difference, averaged across all voxels within the DMFC ROI) for each task and region. For the FM task [24], there was no significant (*p* < 0.05) PPI effect between any of our seed regions and the DMFC ROI (all *p* > 0.2, Figure 2B; left amygdala: *d* = −0.233; right amygdala: *d* = −0.088; sgACC: *d* = 0.162). Therefore, the FM task was not analysed further. 

For the EI task, the average PPI effect for all three seed regions was significant (all *p* < 0.002, Figure 2A; left amygdala: *d* = 0.966; right amygdala: *d* = 0.726; sgACC: *d* = 1.09). For the GC task, the average PPI effect for the sgACC was non-significant (*p* > 0.1), and, therefore, this seed was not analysed further. The GC task PPI effect for both left and right amygdala was significant (both *p* < 0.003) (Figure 2C; left amygdala: *d* = −0.657; right amygdala: *d* = −0.624; sgACC: *d* = −0.162). It is notable that one of the tasks (EI) evoked considerably more robust PPI effects, both within the DMFC ROI (Figure 2) and across the entire brain (see Figure 1). 

The participants showed very high accuracy in both of the tasks analysed: for the EI task, over 91% of button presses were accurate (*SD* = 8.06); for the GC task, over 94% of button presses were accurate (*SD* = 6.33). There was no association between PPI activation and accuracy on the EI (sgACC: *r* = 0.194, *p* = 0.332; left amygdala: *r* = 0.247, *p* = 0.225; right amygdala: *r* = 0.249, *p* = 0.210) or GC task (left amygdala: *r* = 0.172, *p* = 0.382; right amygdala: *r* = 0.291, *p* = 0.126).

For the seed regions resulting in a significant DMFC PPI effect, we conducted ANOVAs, testing for effects of day, run, and the interaction between day and run. There were no significant main effects of day or run for either task, nor any significant interaction between day and run in the EI task, for either the amygdala or the sgACC. However, both regions tested for the GC task (left and right amygdala) showed a day-by-run interaction (left amygdala: *F*(1,27) = 7.12, *p* = 0.013; right amygdala: *F*(1,28) = 6.63, *p* = 0.016), such that activation decreased from run 1 to run 2 on day 1, but *increased* from run 1 to run 2 on day 2 (see Figure 2C). 

### 3.2. Reliability

**Figure 3:**
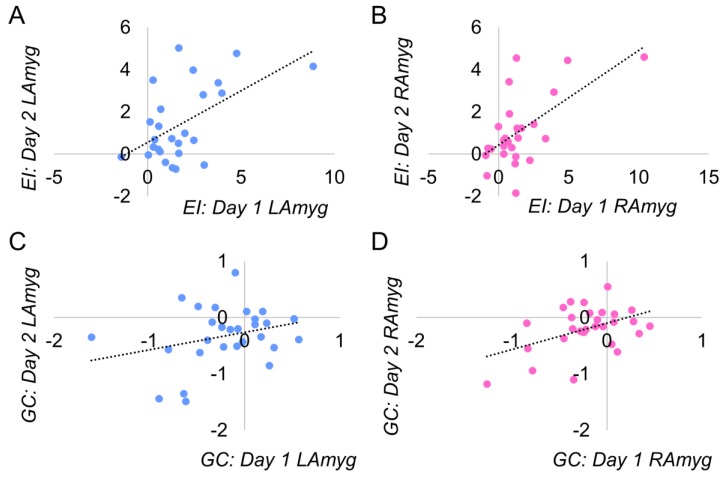
Distribution of PPI effect averaged across days. The regions displayed exceeded our threshold for reliability (ICC > 0.4, see Table 1 for full statistics). The sgACC in the EI task did not exceed our threshold for reliability and is not displayed. EI=emotion identification; GC=gender classification; LAmyg=left amygdala; RAmyg=right amygdala.

We performed ICC analyses to test the within-subject reliability of the PPI effect, across days and runs (two-way fixed effects mixed ICCs; testing for consistency rather than absolute agreement). Table 2 details all reliability statistics computed; scatter plots illustrating the average between-day reliability for the amygdala seed regions are presented in Figure 3.

### 3.3. Emotion Identification

For the EI task, we found good-to-excellent reliability (all ICCs between 0.5 and 0.9) in all between-day and within-day ICC analyses (see Table 1). Note the high between-day reliability averaging across both runs in both left and right amygdala (left: ICC = 0.704 (95%CI = 0.340 to 0.867), *p* = 0.002; right: ICC = 0.738 (95%CI = 0.427 to 0.881), *p* = 0.001), as well as excellent reliability between runs on day 1 for the right amygdala (ICC = 0.832 (95%CI = 0.626 to 0.925), *p* < 0.001) (the same analysis for the left amygdala showed good reliability: ICC = 0.652 (95%CI = 0.224 to 0.844), *p* = 0.005; similarly good reliability was obtained for both amygdalae on day 2). In contrast, the sgACC had only one instance of moderate reliability across all analyses: the within-day reliability on the second day (ICC = 0.408 (95%CI = 0.299 to 0.730), *p* = 0.094). See Figure 3 for average between-day reliability. 

### 3.4. Gender Classification

For the GC task, reliability was more variable. There were two instances of moderate between-day reliability, for the left amygdala (overall, between days, averaging across both runs: ICC = 0.442 (95%CI = 0.206 to 0.742), *p* = 0.068; and between the second runs of both days (ICC = 0.493 (95%CI = 0.095 to 0.765), *p* = 0.042) and for the right amygdala (overall, between days, averaging across both runs: ICC = 0.627 (95%CI = 0.206 to 0.825), *p* = 0.006; and between the first runs of both days (ICC = 0.579 (95%CI = 0.103 to 0.802), *p* = 0.013). However, there were no instances of moderate (or higher) within-day reliability (see Table 2). 

## 4. Discussion

We tested the reliability of amygdala–DMFC and sgACC–DMFC connectivity using a ROI-guided PPI analysis of three emotion-processing tasks. Following initial analyses, only two tasks were analysed for reliability, as one task (face matching) did not show a significant PPI effect using our specific analytic approach. In contrast to our previous results, which showed poor within-subject reliability across these tasks for evoked hemodynamic responses [1], we found good-to-excellent reliability in every amygdala reliability analysis for the emotion identification task. We generally found poorer reliability for sgACC–DMFC task-modulated coupling and mixed reliability for the amygdala–DMFC PPI effect during the gender classification task. 

These findings have particular implications for translational neuroimaging work in anxiety and related disorders. Converging human and animal data have implicated coupling between the DMFC and amygdala in both normal and pathological anxiety [9,16,17,18,19,20]. There are well-known contributions of the amygdala to fear and aversive processing [12,13,14,15] and of the DMFC region in fear appraisal, expression, and pathological anxiety [17,18]. However, there is substantial and bidirectional coupling between the DMFC and the amygdala [19,20]; anxiety behaviours may emerge from changes in the balance of (reciprocal) fronto–amygdala information flow [16]. This coupling has been suggested to represent a common neurobiological phenotype underpinning affective and anxiety disorders (within the ‘negative valence systems’ construct of the Research Domain Criteria) [16], making its investigation as a putative biomarker essential.

Our target ROI was taken directly from the anxiety literature [11] (ROI available at https://figshare.com/authors/Oliver_Robinson/568652). This enabled us to test whether task-related amygdala–DMFC coupling was sufficiently reliable to justify its exploration as a biomarker of treatment response, as previously suggested [9]. We provide support for the use of one of our tasks (emotion identification) in this endeavour and weaker support for another (gender classification). 

It is notable that one of the tasks (emotion identification) evoked very robust PPI effects, both in the ROI (see Figure 2) and the brain as a whole (see Figure 1). One of the tasks (face matching) showed no significant PPI effect in the DMFC ROI, and for the other (gender classification), the PPI effect was more modest. The tasks differed in several key aspects which could have contributed to these differences: the emotion identification task was the only one that required explicit emotional labelling; one possibility is that such labelling evokes greater prefrontal–subcortical coupling than the incidental processing of emotional faces. Indeed, somewhat surprisingly, while we identified a significant increase in amygdala–DMFC covariation during the emotion identification task (relative to fixation baseline), this covariation was significantly decreased during the gender classification task. 

### Limitations and Future Directions

There are a number of important limitations to our findings. First, we conducted a very specific type of connectivity analysis (PPI), so our cautiously optimistic finding of good within-subject reliability for PPI with the amygdala, particularly in the emotion identification task, may not apply to data analysed using a different method. Others have reported similarly high reliability estimates of so-called “resting-state” connectivity measures, ranging from moderate to large [33,34,35], though these differ widely depending on the methodology used and the measure tested [35]. It is also worth noting that at least some resting-state reliability estimates may be artificially inflated because of the extremely high reliability of physiological noise (cardiac and respiratory) [36], while this is much less of a concern for our PPI analyses (as such, physiological noise is presumably similar between the task conditions). Second, it will be essential to test the reliability of activation and PPI effects in clinical samples, who may differ in relevant aspects from our sample of healthy controls. Finally, in larger samples than ours, it will also be important to perform more exploratory analyses of PPI between these seed regions and the rest of the brain. In this study, we restricted ourselves to a single DMFC ROI (and a single fMRI contrast for each task, as in our previous work [1]). This allowed us to constrain our hypotheses to avoid false positives, which could arise from a combination of multiple testing and low statistical power (common in PPI) [28]; however, this approach precludes inference about other regions.

## 5. Conclusions

We report good within-subject reliability of amygdala–DMFC PPI using emotion-processing tasks, both between days (in two tasks) and within-day (between runs, in one task). This suggests that PPI may have more utility as a ‘biomarker’ to predict treatment outcome than task-related hemodynamic responses, which (in this dataset at least), showed very poor within-subject reliability [1]. Functional connectivity has been implicated as a possible ‘biomarker’ across a number of studies and treatment modalities, including pharmacological [5,6,37], psychological [8,37], and brain stimulation [38]. It is certainly a compelling suggestion that regional coupling implicated in a disorder could be tested as a predictor of treatment response. Our finding of relatively good within-subject reliability tentatively supports testing this ambitious proposal in future translational work. 

## Figures and Tables

**Table 1:** Task characteristics.

Characteristic	Emotion Identification (EI)	Face Matching (FM)	Gender Classification (GC)
Task duration	4:03	5:55	6:24
Task design	Event-related	Blocked	Blocked
Regressors of interest	Happy; fearful; neutral	Faces; shapes	Happy; fearful; neutral
Instruction	Explicit (match emotion)	Implicit (match face or shape to test stimuli)	Implicit (classify gender of face)
Regressors of no interest	6 movement parameters	6 movement parameters	6 movement parameters + errors
Contrast	Faces > fixation	Faces > shapes	Faces > fixation

Design and analysis characteristics of the three emotional processing tasks used.

**Table 2:** Results from intraclass correlation coefficient (ICC) analysis of PPI for the EI and GC tasks, extracted from the DMFC region of interest.

Task	Reliability	LAmyg ICC (95% CI)	*p*-value	RAmyg ICC (95% CI)	*p*-value	sgACC ICC (95% CI)	*p*-value
*EI*	Between-day (both runs)	**0.704** (0.340 to 0.867)*	0.002	**0.738** (0.427 to 0.881)*	0.001	0.121 (−0.928 to 0.600)	0.372
	Between-day (run 1)	**0.585** (0.074 to 0.814)*	0.016	**0.765** (0.485 to 0.893)*	<0.001	0.167 (−0.828 to 0.620)	0.323
	Between-day (run 2)	**0.573** (0.047 to 0.808) *	0.019	**0.555** (0.008 to 0.801)*	0.024	0.017 (−1.158 to 0.552)	0.483
	Within-day (day 1)	**0.652** (0.224 to 0.844)*	0.005	**0.832** (0.626 to 0.925)*	<0.001	0.314 (−0.504 to 0.688)	0.171
	Within-day (day 2)	**0.768** (0.482 to 0.896)*	<0.001	**0.699** (0.339 to 0.863)*	0.002	**0.408** (0.299 to 0.730)*	0.094
*GC*	Between-day (both runs)	**0.442** (−0.206 to 0.742)*	0.068	**0.627** (0.206 to 0.825)*	0.006	Not analysed	
	Between-day (run 1)	0.071 (−1.01 to 0.570)	0.425	**0.579** (0.103 to 0.802)*	0.013	Not analysed	
	Between-day (run 2)	**0.493** (−0.095 to 0.765)*	0.042	0.326 (−0.435 to 0.684)	0.151	Not analysed	
	Within-day (day 1)	0.143 (−0.852 to 0.603)	0.346	0.146 (−0.818 to 0.599)	0.339	Not analysed	
	Within-day (day 2)	0.171 (−0.792 to 0.616)	0.315	−0.319 (−1.810 to 0.381)	0.766	Not analysed	

ICCs, their 95% confidence intervals (CI) and associated *p*-values are presented for each seed region analysed, for each task; * indicates ICCs exceeding 0.4 (these ICCs are depicted in bold), the lower bound for moderate reliability. EI=emotion identification; GC=gender classification; LAmyg=left amygdala; RAmyg=right amygdala; sgACC=subgenual anterior cingulate cortex; DMFC=dorsomedial frontal cortex; CI=confidence interval.
